# Libel and Slander as Affecting Physicians

**Published:** 1898-10

**Authors:** 


### Libel and Slander as Affecting Physicians.

---

Henry Leffman contributing to the *Philadelphia Polyclinic*, a syllabus of the law relating to this important subject:

Libel consist in the utterance of any communication otherwise than by oral speech unjustifiably accusing private individuals, officials or governments of anything tending to make them ridiculous or injure them in reputation or public esteem. Slander is an oral statement unjustifiably accusing a person of a crime, a loathsome disease, incapacity, or dishonesty, or of any fault which tends to injure the person or his business. The courts have decided that an accusation may be slanderous or not according to the vocation of the accused. To accuse a physician of general professional ignorance or malpractice is actionable *per se*, but to state that in a special case he was at fault is not slanderous, unless special damage is proved. A retired physician, since he no longer gains his living by his profession, may be accused with impunity of what would "slander" a man in actual practice. Slanderous words uttered in one State may not be actionable in another State unless proved to be so also in the place uttered. A person uttering slander to a second party who repeats it to the detriment of an individual may escape responsibility if the damages result from the utterance of the second party and not from that of the originator. In case of libel, any accusation holding a person up to scorn or ridicule, whether professionally or as a private person, is actionable. A physician who attempts to achieve notoriety by puffing himself cannot recover damages from those who further his attempts. It is slander to falsely attribute a contagious disease to a person, unless a statement was necessary and there was a mistaken diagnosis. A physician condemning any article used in medical practice is liable to the manufacturer, if the said physician's statement be incorrect.—*New England Medical Monthly.*

